# Effective Antibiotics against ‘*Candidatus* Liberibacter asiaticus’ in HLB-Affected Citrus Plants Identified via the Graft-Based Evaluation

**DOI:** 10.1371/journal.pone.0111032

**Published:** 2014-11-05

**Authors:** Muqing Zhang, Ying Guo, Charles A. Powell, Melissa S. Doud, Chuanyu Yang, Yongping Duan

**Affiliations:** 1 Guangxi University, Nanning, 530004, China; 2 Institute of Food and Agricultural Science-Indian River Research and Education Center, University of Florida, Fort Pierce, FL 34945, United States of America; 3 United States Department of Agriculture-Agriculture Research Service-United States Horticultural Research Laboratory, Fort Pierce, FL 34945, United States of America; UMBC, United States of America

## Abstract

Citrus huanglongbing (HLB), caused by three species of fastidious, phloem-limited ‘*Candidatus* Liberibacter’, is one of the most destructive diseases of citrus worldwide. To date, there is no established cure for this century-old and yet, newly emerging disease. As a potential control strategy for citrus HLB, 31 antibiotics were screened for effectiveness and phytotoxicity using the optimized graft-based screening system with ‘*Candidatus* Liberibacter asiaticus’ (Las)-infected citrus scions. Actidione and Oxytetracycline were the most phytotoxic to citrus with less than 10% of scions surviving and growing; therefore, this data was not used in additional analyses. Results of principal component (PCA) and hierarchical clustering analyses (HCA) demonstrated that 29 antibiotics were clustered into 3 groups: highly effective, partly effective, and not effective. In spite of different modes of actions, a number of antibiotics such as, Ampicillin, Carbenicillin, Penicillin, Cefalexin, Rifampicin and Sulfadimethoxine were all highly effective in eliminating or suppressing *Candidatus* Liberibacter asiaticus indicated by both the lowest Las infection rate and titers of the treated scions and inoculated rootstock. The non-effective group, including 11 antibiotics alone with three controls, such as Amikacin, Cinoxacin, Gentamicin, Kasugamycin, Lincomycin, Neomycin, Polymixin B and Tobramycin, did not eliminate or suppress Las in the tested concentrations, resulting in plants with increased titers of Las. The other 12 antibiotics partly eliminated or suppressed Las in the treated and graft-inoculated plants. The effective and non-phytotoxic antibiotics could be potential candidates for control of citrus HLB, either for the rescue of infected citrus germplasm or for restricted field application.

## Introduction

Three species of the fastidious, phloem-residing, gram-negative bacteria, ‘*Candidatus* Liberibacter asiaticus’ (Las) [1], ‘*Ca.* L. africanus’ (Laf) [2] and ‘*Ca.* L. americanus’ (Lam) [3] are the causal agents of huanglongbing (HLB, also known as greening), one of the most devastating diseases of citrus. Both Las and Lam are transmitted by *Diaphorina citri* Kuwayama, while Laf is transmitted by *Trioza erytrea* (Del Guercio) [4]. Citrus HLB was first reported in China in 1919, but most likely originated in Taiwan in the 1870s [1], [5]. It was estimated that more than 100 million infected citrus trees have been destroyed by the disease throughout Asia, while more than four million trees have been eliminated in Brazil since the first report in São Paulo in 2004 [6]. In the U.S., HLB was first discovered in August 2005 in South Florida, and currently is endemic in all 34 citrus-producing counties in Florida. HLB has caused an estimated losses of $1.3 billion in direct revenue and $3.6 billion in indirect revenue [7]. The HLB-associated bacteria infect all cultivated citrus species and relatives. The recommended management strategy for HLB includes chemical control to reduce psyllid populations, removal of infected trees to eliminate new sources of bacterial inoculum and production of pathogen-free nursery plants [8]. Although there are no practical methods for the control of HLB in commercial groves [1], graft-based chemotherapy [9], shoot tip grafting [10], [11], thermotherapy [12]–[15], vitrification-cryopreservation [16] and antibiotics [13] have been successfully used for HLB therapy in the greenhouse settings.

With the finding that prokaryotic organisms were associated with HLB [1], an effort to control disease in existing orchards was made by injecting trees with antibiotics in several countries or regions, including China, India and South Africa, [10], [13], [17]–[21]. Several researchers reported initial success in reversing the symptoms of HLB [22]–[24]. In our previous studies, penicillin applied alone or in combination with streptomycin (PS) was shown to be effective in eliminating Las, and PS, when compared to administering either antibiotic separately, provided a therapeutically effective level of control for a greater period of time [9].

Because of unprecedented epidemics of citrus HLB in Florida and other citrus growing regions in the world, chemotherapy, including the use of antibiotics against Las, is urgently needed for the survival of the Florida citrus industry. Screening and development of a bactericide or other curative product is one of the most promising approaches for the control of HLB. In this study, we have evaluated 31 antibiotics for their effectiveness against Las and their phytotoxicity to citrus using the optimized graft-based chemotherapy method [9]. A number of effective antibiotics, especially agricultural antibiotics have been identified for further evaluation in field trials.

## Materials and Methods

### Chemicals and preparation of working concentrations

In this study, antibiotics and their concentration used for screening were selected based on literature review [43] and/or from suggestions from the Solvers of the InnoCentive™ group who had partnered with the Citrus Research and Development Foundation (CRDF), Florida, USA (Table 1). This group solicited a call throughout the world for suggestions of chemicals that may combat against Las infection. Based on these suggestions, 31 antibiotics were chosen to screen for phytotoxicity and effectiveness against Las. Two agricultural antibiotics zhongshengmycin and validoxylamine A were purchased from Fujian Kaili Bio-Product Co. Ltd (Fuzhou, Fujian, China). All other antibiotics were obtained from Sigma-Aldrich, Co. (St. Louis, MO, USA). Antibiotics were freshly prepared by dissolving the appropriate amount in solvent as listed in Table 1 unless noted below. Rifaximin, sulfamethoxazole and chloramphenicol were first dissolved in 1 ml of ethanol and then adjusted to 1,000 ml of the final concentration with water, while cinoxacin was initially dissolved in 1 ml of dimethyl sulfoxide (DMSO) and then the final dilution was made up to 1,000 ml with water. Four antibiotics (PEN, VA, ZS and KSM) were further evaluated to confirm the efficacy against Las bacteria at three different concentrations of 10 mg/L, 100 mg/L and 1,000 mg/L.

**Table 1:** Antibiotics screened for the control of citrus Huanglongbing and the concentrations used.

Code	Chemical compounds	Antibiotic classes	Working conc. (mg/L)	Solvent
ACT	Actidione	Agro-antibiotics	25	water
VA	Validoxylamine A	Agro-antibiotics	100	water
ZS	Zhongshengmycin	Agro-antibiotics	100	water
AMK	Amikacin sulfate	Aminoglycoside	100	water
GAT	Gentamicin sulfate	Aminoglycoside	100	water
HYG	Hygromycin B	Aminoglycoside	150	water
KAN	Kanamycin sulfate	Aminoglycoside	100	water
KSM	Kasugamycin hydrochloride	Aminoglycoside	100	water
NEO	Neomycin hydrate trisulfate	Aminoglycoside	50	water
SPT	Spectinomycin dihydrochloride pentadrate	Aminoglycoside	20	water
STR	Streptomycin sulfate	Aminoglycoside	100	water
TOB	Tobramycin	Aminoglycoside	20	water
AMP	Ampicillin sodium	Beta-Lactam	100	water
CAR	Carbenicillin disodium	Beta-Lactam	100	water
PEN	Penicillin G potassium	Beta-Lactam	100	water
CEF	Cefalexin	Cephalosporins	100	water
VAN	Vancomycin hydrochloride	Glycopeptide	40	water
LIN	Lincomycinhydrocloride	Lincosamide	100	water
CYS	Cycloserine	Oxazolidinones	50	water
RIF	Rifamycin sodium	Ansamycin	50	water
RIM	Rifampicin	Ansamycin	50	water
RIX	Rifaximin	Ansamycin	50	ethanol
COL	Colistinmethanesulfonate sodium	Polypeptide	20	water
PMB	Polymixin B sulfate	Polypeptide	300	water
CIN	Cinoxacin	Quinolone	300	DMSO
CIP	Ciprofloxacin hydrochloride	Quinolone	300	water
SDX	Sulfadimethoxine sodium	Sulfonamides	100	water
SMZ	Sulfamethoxazole	Sulfonamides	100	ethanol
STZ	Sulfathiazole sodium	Sulfonamides	100	water
CHL	Chloramphenicol	Tetracycline	30	ethanol
OXY	Oxytetracycline hydrochloride	Tetracycline	100	water

### Graft-based evaluation assay

To identify the effectiveness of a chemical against Las and its phytotoxicity to citrus, antibiotics were evaluated using the previously published graft-based chemotherapy [9]. Briefly, HLB-affected budsticks were sampled from severely HLB-affected rough lemons (*Citrus limonum* ‘Lemon #76’) at the USDA-ARS-USHRL farm in Fort Pierce, FL and confirmed positive for Las by real-time qPCR [9], [25]. The budsticks were individually soaked in antibiotic solutions as listed in Table 1 (total 45 scions per each antibiotic at one concentration) overnight in a fume hood under ventilation and continuous fluorescent light. Water, 0.1% of DMSO, and 0.1% of ethanol were used as negative controls, respectively. Each antibiotic-soaked budstick was cut into 2-buds scions and grafted onto an individual two-year-old healthy grapefruit (*Citrus paradisi* ‘Duncan’) rootstocks and covered with plastic tape for three weeks. To improve scion growth, new flush from the rootstocks was removed immediately after grafting and only allowed to grow after the scion had flushed. All experimental plants were grown at 25°C±2°C under shade in an insect-proof greenhouse.

### Monitoring of Las infection and tree health

The effectiveness of the antibiotic against Las was determined by measuring the titer of Las in both the grafted scion and the rootstock using qPCR. Briefly, five leaves were sampled from scions (rough lemon) and rootstocks (grapefruit) four months after grafting (120 DAT), and then again two months later (180 DAT). The leaves were washed in tap water and then rinsed three times with sterile water. The midribs of the leaves were excised, frozen in liquid nitrogen, and stored at −80°C for further processing. The midribs of five leaves from each sample were pooled together and used for DNA extraction and subsequent qPCR analysis as described previously [9], [25].

Genomic DNA was extracted from 0.1 g of tissue (fresh weight) of leaf midribs using Qiagen DNeasy Plant Mini Kit (Qiagen, Valencia, CA) according to the manufacturer. Real-time quantitative PCR (qPCR) was performed with primers and probes (HLBasf, HLBr, and HLBp) for the ‘*Ca.* L. asiaticus’ bacterium (25, 27) using ABI PRISM 7500 sequence detection system (Applied Biosystems, Foster City, CA) in a 20-μl reaction volume consisting of the following reagents: 300 nM (each) target primer (HLBasf and HLBr), 150 nM target probe (HLBp), and 1× TaqMan qPCR Mix (Applied Biosystems). The amplification protocol was 95°C for 20 s followed by 40 cycles at 95°C for 3 s and 60°C for 30 s. All reactions were performed in triplicate and each run contained the same negative (DNA from healthy plant) and positive (DNA from ‘*Ca.* L. asiaticus’-infected plant) control. Data were analyzed using the ABI 7500 Fast Real-Time PCR System with SDS software. The resulting cycle threshold (Ct) values were converted to the estimated bacterial titers using the grand universal regression equation *Y*  =  13.82–0.2866*X*, where *Y* is the estimated log concentration of templates and *X* is the Ct values from qPCR, as described by Li et al. (25). As in the previous report (30), plants tested negative by nested PCR with primer sets OI1/OI2c and CGO3f/CGO5r [26], [27] for ‘*Ca.* L. asiaticus’ when the Ct values were>36.0, which is equivalent to the estimated bacterial titers of <1,060 cells/g of plant tissue. Therefore, the scion infection (%) was defined as the number of Las-infected scions with threshold cycle (Ct) values below 36.0 divided by the number of growing scions. The Las transmission (%) was defined as the number of the grafted rootstocks that tested Las-positive by qPCR with Ct values less than 36.0 out of the total grafted rootstocks. Data correlates with the absence of HLB-like symptoms in the inoculated plants (48).

The phytotoxicity was determined by the percentage of the scions that survived and grew. The percentage of scion survival was calculated by dividing the number of scions that survived by the total number of grafted scions. The scion growth (%) was defined as the number of scions that had newly emerging leaves or flushes out of the total number of grafts.

### Data analysis

Variance analysis was carried out to individually compare antibacterial activity and phytotoxicity of the antibiotic treatments using the SAS/STAT procedure ANOVA. The percentage of scion infection and Las transmission were transformed with the Arcsine square root such that the transformed errors were normally distributed for ANOVA analysis. Differences among antibiotic treatment levels were assessed by Duncan's multiple range tests at Pr ≤ 0.05 (SAS V.9.1, SAS Institute, NC, USA). Further evaluations were conducted to permit integrated comparisons between antibiotics by simultaneously considering all antibacterial and phytotoxic effects. Mean data from all 45 scions in each treatment at the same sampling time were used for principal component analysis (PCA) and hierarchical clustering analysis (HCA) by SAS/STAT procedure PRINCOMP and CLUSTER, respectively. All data were standardized before analysis (the mean of the values for each variable was subtracted from each variable value and the result was divided by the standard deviation of the values for each variable). Then, PCA transformed the original measured variables into new uncorrelated variables called principal components [28]. HCA was applied to the standardized data to investigate similarities between different antibiotics in antibacterial activity. Euclidean distance for HCA between antibiotics was calculated using a defined metric. In the single linkage method, the distances or similarities between two clusters, A and B, was defined using an unweight pair group method with arithmetic mean (UPGMA) [29]. The stepwise discriminant analysis (SDA) was used to select the variables most useful in discriminating the samples from the above HCA clusters using the SAS/STAT procedure STEPDISC. In brief, six variables of antibacterial activities, including scion infection percentage (SI), Las transmission percentage (LT), Ct value in scions (CTS1 and CTS2) and rootstocks (CTR1 and CTR2) of the graft-inoculated plants at 120 DAT and 180 DAT, respectively, were evaluated at each step of the SDA process. The variable within the model, which contributed least to the model as determined by the Wilk's Lambda method and the significance level of F test at Pr≤0.05, was removed from the model. Likewise, the variable outside the model that contributed most to the model was added. When no more steps could be taken, the number of variables in the model was reduced to its final form. The final variables in turn were subjected to discriminant analysis to develop models for discriminating the antibacterial efficiency of antibiotics. Groups were separated using Tukeys' test and considered significantly different at Pr≤0.05 (SAS V.9.1, SAS Institute, NC, USA).

## Results

### Phytotoxicity of chemicals

Significant phytotoxicity resulted from treatment with ACT and OXY (Pr≤0.05). Less than 13.6% and 6.3% of scions treated, respectively survived; whereas, a higher percentage of the scions treated with the remaining antibiotics and control solvents survived (from 57.1% to 100%) and produced more flushes and/or new leaves. Due to less than 15.0% of scions surviving and those living produced little to no new flush (5 and 6.3% respectively), ACT and OXY was discarded from further analysis. Scions soaked in COL, LIN, SPT, and VAN had a 100% survival rate and more than 50.0% of scion growth. KSM, RIF and VAN had the highest percentages of scion growth (more than 85.0%). The control solvents resulted in approximately 91.0% of the scions surviving (Table 2).

**Table 2:** Efficacy of antibiotics against Las bacterium.

Antibiotics	Scion survival (%)	Scion grown rate (%)	Scion infected (%)	Las transmission (%)	Ct value in Scion	Ct value in Rootstock	SDA^y^	
							Group	posteriori probability
ACT	13.6	5±7.1	0±0	0±0	39.2±1.2	38.7±1.4	ND	ND
AMK	83.3	52.8±3.9	85.0±21.2	77.8±0	30.4±5.2	27.9±6.9	III	1
AMP	96.4	73.2±2.5	0±0	0±0	39.7±0.7	39.6±0.8	I	1
CAR	75	50±0	0.0±0	0±0	38.0±3.0	37.4±2.7	I	1
CEF	77.3	15.9±3.2	29.2±5.9	50.0±6.4	38.4±2.5	33.9±7.0	I	1
CHL	96.2	75±0	80.6±3.9	39.7±9.1	31.1±5.6	33.4±6.7	II	1
CIN	68.2	18.9±5.5	80.0±28.3	71.4±1.9	27.5±7.3	27.1±7.8	III	1
CIP	85	18.6±5.1	75.0±35.4	11.1±15.7	33.0±5.80	38.3±3.0	II	1
COL	100	61.4±16.1	69.4±0.9	63.2±37.9	30.7±5.6	31.3±7.3	III	0.68
CYS	92.3	65.4±21.8	58.4±15.3	50.0±27.2	33.0±6.1	32.8±6.9	II	1
GAT	80	47.5±10.6	100±0	80.0±14.1	25.3±3.5	29.5±5.6	III	1
HYG	87.5	68.3±9.5	50.7±16.7	47.8±8.6	34.0±5.7	33.9±6.7	II	1
KAN	61.5	25±2.7	61.9±6.7	50.0±5.4	32.8±6.1	33.7±6.5	II	0.99
KSM	95.8	89.6±14.7	87.9±5.3	79.2±5.9	28.7±4.8	28.6±7.3	III	1
LIN	100	52.1±8.8	81.8±25.7	91.7±0.0	28.6±5.4	24.7±5.8	III	1
NEO	75	39.2±2.4	88.9±15.7	68.6±32.7	26.7±5.5	29.4±7.4	III	1
OXY	6.3	6.3±0	0±0	0±0	38.2±2.5	38.9±1.3	ND	ND
PEN	75	25±14.1	0±0	0±0	37.6±3.0	38.8±2.0	I	1
PMB	90.9	62.5±17.7	75.5±21.9	85.5±7.7	31.5±5.2	28.8±6.0	III	1
RIF	91.7	85.4±8.8	40.7±31.8	45.8±5.9	35.5±5.7	34.2±5.50	II	1
RIM	86.4	37.5±3.5	20.5±11.4	16.1±8.6	38.1±3.0	36.2±5.6	I	0.99
RIX	58.3	33.3±5.9	52.4±26.9	50.0±11.8	33.9±7.0	32.3±8.2	II	0.99
SDX	75	52.5±10.6	27.8±7.9	10±0	36.0±5.1	38.1±4.1	I	1
SMZ	90	45±7.1	81.3±8.8	28.8±12.4	30.7±4.9	34.6±7.0	II	1
SPT	100	50.1±4.9	65.7±8.1	41.4±2.0	32.3±5.6	33.1±7.4	II	1
STR	95	50±0	80.0±0	65.0±21.2	31.7±5.1	30.8±6.8	III	0.50
STZ	65	35±7.1	47.9±20.6	21.1±1.6	35.0±4.1	36.7±5.3	II	0.86
TOB	95	25±0	100±0	85.0±7.1	25.4±3.7	26.8±6.7	III	1
VA	95.3	35.2±20.7	85.5±8.7	40.2±18.1	30.4±4.5	34.9±5.8	II	0.98
VAN	100	90±0	75.0±35.4	60.0±42.4	30.8±6.0	31.5±8.8	III	0.88
ZS	57.1	18.3±1.5	61.1±32.3	44.3±5.3	32.5±5.4	34.4±6.1	II	1
Water	91.3	40.3±4.0	91.9±11.5	100.0±0	29.8±5.1	23.8±3.6	III	1
DMSO	91.7	45.2±3.6	50±2.1	93±3.8	25±2.8	25.2±3.1	III	1
Ethanol	90.9	50.4±4.8	60±5.1	97.2±4.6	26.5±0.7	25.8±1.9	III	1

Transmission efficiency of Las along with scion survival (%) and scion infection (%) and the Ct value in the scion and rootstock in the inoculated plants when grafted with Las-infected scions (Ct value = 25.7±2.1) treated with antibiotics.

y SDA: Stepwise discriminant analysis; The group is classified by SDA.

### Antibacterial activities of the antibiotics against Las bacteria

**Figure 1:**
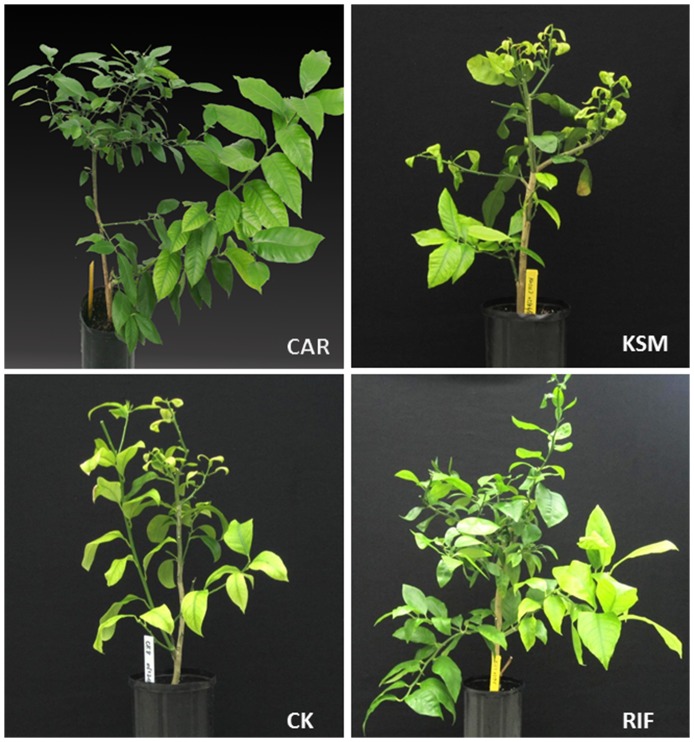
Huanglongbing (HLB)-affected grapefruit (‘Duncan’) plants with graft-inoculation of Las-infected lemon scions treated with different antibiotics. **I-CAR**: Highly effective compounds; **II-RIF**: Partly effective compounds; **III-KSM**: non-effective compounds; **CK**: water control. Photograph was taken 6 months after graft inoculation. Typical HLB symptom of leaf curl and corky veins on grapefruit rootstock and blotchy mottle or yellow shoots on leaves of lemon scion were apparent.

Variance analysis showed that there were significant effects of the antibiotics (Pr =  0.000) on Las titers (Ct values), percentage of infected scions and Las transmission (%) in the fixed model. The inoculated plants grown from Las-infected scions soaked in beta-lactam antibiotics (AMP, PEN and CAR) showed a marked reduction (Pr≤0.05) in Las, resulting in Ct values greater than 36.0, with an estimated bacterial titer of less than 1,000 cells/g of tissue. Based on the Ct cut off at 36.0, the scions and rootstocks from the inoculated plants grafted with HLB-affected scions treated with beta-lactam antibiotics were no longer infected after six months, which indicated that these antibiotics eliminated most if not all of the Las bacteria in HLB-affected scions. Scions soaked in RIM and SDX also showed a reduction in average Las titer with Ct values of 38.1 and 36.0, respectively. However, Las was transmitted to 16.1% and 10.0% of the rootstocks, respectively. AMK, CIN, GAT, KSM, LIN, PMB and TOB, were not as effective in suppressing Las as more than 70.0% of the rootstocks were infected (Table 2). The control solvents, such as 0.1% of DMSO, 0.1% of ethanol and water, had no significant effects on Las titers in the inoculated rootstock (Ct value from 23.8 to 25.8) and citrus scions (Ct value from 25.0 to 29.8) (Pr = 0.281). The plants grafted with control solvent-soaked Las positive scions had typical HLB symptoms, such as yellow shoots and/or corky leaves on the rootstocks, or blotchy mottled leaves on the scions as did scions treated with KSM and other non-effective compounds whereas no HLB-like symptoms were displayed in the scions and rootstocks of the inoculated plants grafted with scions soaked in CAR or other highly effective compounds (Fig. 1).

### Principal component analysis (PCA)

**Figure 2:**
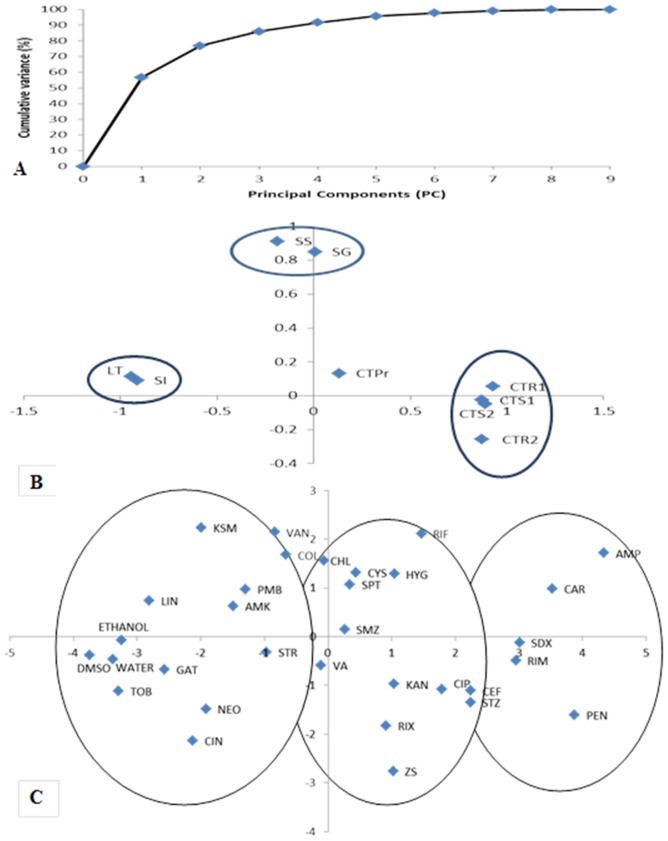
Principal component analysis (PCA) plots. A) Cumulative variance; B) Loading plots for different variables on PC_1_ (antibacterial activity) and PC_2_ (phytotoxicity). CTPr: Ct before treatment of infected scion; CTS-1 and CTR-1: Ct value in the scion and rootstock of the graft-inoculated at DAT 120, respectively; CTS-2 and CTR-2: Ct value in the scion and rootstock of the graft-inoculated at DAT 180, respectively; SI36 and LT36: Scion infection percentage (SI) and Las transmission rate (LT) were calculated when Ct value was cut off at 36.0, respectively; SS and SG: Scion survival and scion growth, respectively; C) PCA scores plot for different antibiotics on PC_1_ (antibacterial activity) and PC_2_ (phytotoxicity).

PCA was applied to the data set of 29 tested antibiotics (excluding ACT and OXY) after standardization as described in the methods. PC_1_ accounted for 56.7% of the total variance in the data set while PC_2_ explained 20.1% (Fig. 2A). The contribution of each variable, their relationships and the resulting principal components were illustrated in Fig. 2B. The Ct values observed in the post-treatment samples (scion and rootstock at DAT 120 [CTS-1 and CTR-1, respectively] and at DAT 180 [CTS-2 and CTR-2, respectively]) contributed primarily to PC_1_, as did the data on the percentage of scion infection (SI) and Las transmission (LT) but with opposite values. The scion survival (SS) and growth percentages (SG) contributed primarily to PC_2_. The Ct values of pre-treated scions (CTPr) contributed almost equally to PC1 and to PC2 (Fig. 2B). A number of observations were made from the antibiotic score plot for PC_1_ *versus* PC_2_ (Fig. 2C). Firstly, 16 antibiotics, such as AMP, CAR, PEN, SDX and RIM, were located in the right quadrants while the other antibiotics and controls (water, DMSO and ethanol) were situated in the left quadrants. Secondly, 14 antibiotics, such as AMP, CAR and VAN, were located on the upper quadrants of the plot while the other 15 antibiotics and 3 controls were located in the lower quadrants. The antibiotics in the right quadrants, *e.g.*, AMP, CAR, PEN, SDX and RIM, were effective in eliminating or suppressing Las, while antibiotics on the upper quadrants, *e.g.* AMP, CAR and VAN, were less phytotoxic to citrus.

### Hierarchical cluster (HCA) and stepwise discriminant analysis (SDA)

**Figure 3:**
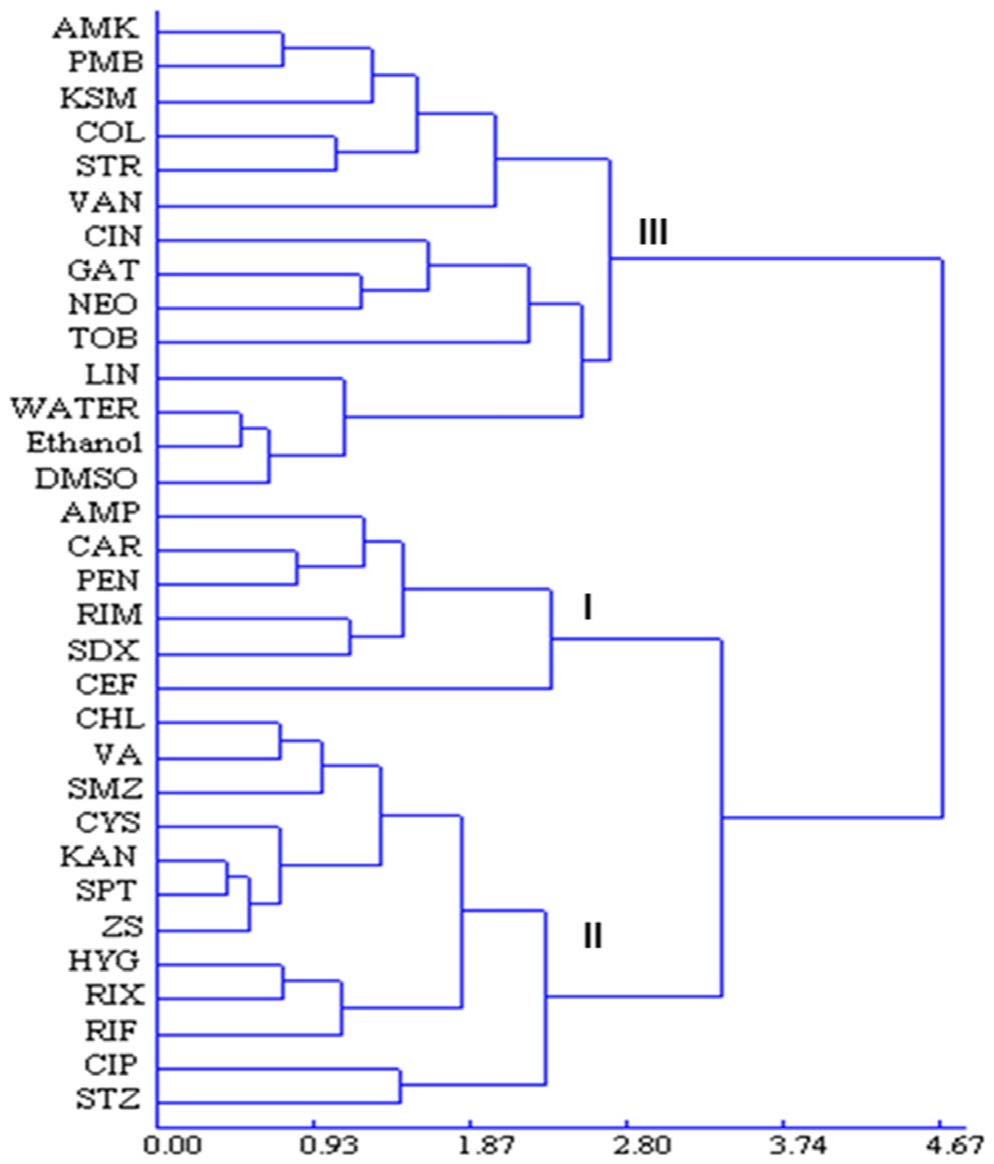
Dendrogram of the hierarchical cluster analysis for antibacterial activity against Las with 29 antibiotics.

In hierarchical cluster analysis, antibiotics were classified on the basis of scion infected percentage, Las transmission percentage, and Ct values of scions and rootstocks in the inoculated plants, without considering the information about antibiotic class. The results obtained by HCA were shown as a dendrogram (Fig. 3) in which three well-defined clusters are visible in terms of their similarities. A group of antibiotics (group I), composed of six compounds, AMP, CAR, PEN, CEF, RIM and SDX, was clearly discernible. These antibiotics were associated with high antibacterial activities against Las, resulting in the lowest percentages of Las-infected scions and transmission of Las bacterium into the rootstocks, and the lowest bacterial titers in the scions and rootstocks of the graft-inoculated plants (Table 2). Group III consisting of 11 antibiotics (AMK, PMB, KSM, COL, STR, VAN, CIN, GAT, NEO, TOB and LIN) and the three solvent controls (water, DMSO and ethanol) had the lowest antibacterial effects. The highest percentage of infected scion and Las transmission and the bacterial titers (lowest Ct values) were observed in the plants grafted with scions soaked in these group III chemicals (Table 2). Thus, these compounds were not effective in eliminating or suppressing Las bacterium. Group II, which included CHL, VA, SMZ, CYS, KAN, SPT, ZS, HYG, RIX, CIP, STZ and RIF, partly eliminated or suppressed Las when compared to Group I and Group III data (Table 2 and Table 3).

**Table 3:** Antibiotics classification of antibacterial activity against Las bacterium by hierarchical cluster analysis.

Variables		Group I	Group II	Group III
Scion survival (%)		80.9±8.0 a^y^	81.7±15.5 a	89.8±9.4 a
Scion growth (%)		42.4±18.9 a	46.2±21.7 a	51.8±19.4 a
Scion infection (%)		17.4±10.6 c	63.4±13.9 b	86.5±9.8 a
Las transmission (%)		16.9±16.2 c	39.2±12.0 b	80.5±9.8 a
	CTPr	25.9±2.7 a	26.1±1.9 a	25.1±1.9 a
	CTS1	37.0±1.70 a	33.3±2.2 b	29.5±2.8 c
**Ct value**	CTR1	37.7±1.7 a	35.2±1.0 b	29.1±3.7 c
	CTS2	38.3±1.7 a	32.3±1.4 b	27.2±3.0 c
	CTR2	37.0±2.2 a	33.6±2.7 b	26.4±2.4 c
**Compounds included**		AMP, CAR, PEN, CEF, RIM, SDX	CHL, VA, SMZ CYS, KAN, SPT ZS, HYG, RIX CIP, STZ, RIF	AMK, PMB, KSM, COL, STR, VAN, CIN, GAT, NEO, TOB, LIN, WATER, DMSO, ETHANOL
**Group Classification**		Highly effective	Partly effective	Non-effective

Group means and standard error of transmission efficiency of ‘*Candidatus* Liberibacter asiaticus’ (Las) along with scion infection (%) and the Ct value of the pre-treated scions (CTPr) and post-inoculated scions and rootstocks after grafted with Las-infected scions treated with antibiotics.

y Different letter by group indicated the significance at 0.05 level. Notes: CTPr, mean Ct value from the grafted scions before treatment. CTS1 and CTR1: Mean Ct value in the scion and rootstock of the graft-inoculated plants at DAT120, respectively; CTS2 and CTR2: Mean Ct value in the scion and rootstock of the graft-inoculated plants at DAT180, respectively.

The results from stepwise discriminant analysis (SDA) showed that scion infected percentage (SI), Las transmission rate (LT) and Ct values in the scions (CTS1 and CTS2) of the inoculated plants were selected for the discriminant function based on the Wilk's Lambda and F value (Pr = 0.00000, *Chi* = 76.4) (Table 4). By using the above four variables as predictors, 100% of the antibiotics were correctly classified into HCA-clustered groups from all six variables. Twenty-eight out of 32 antibiotics and controls (except COL, STR, STZ and VAN) were correctly classified as that the overall posteriori probability was more than 98.0%.

**Table 4:** Selected variables of antibacterial activity by stepwise discriminant analysis at *Chi* = 76.4 and *P* = 0.00000.

Variables	Wilks Lambda	F value	Selected (Y/N)
CTS1^a^	0.708	5.37	Y
CTR1^a^	0.917	1.13	N
CTS2^b^	0.853	2.25	Y
CTR2^b^	0.947	0.71	N
SI^c^	0.519	12.05	Y
LT^d^	0.493	13.36	Y

a CTS_1_ and CTR_1_: Ct value in the scion and rootstock of the graft-inoculated at DAT 120, respectively.

b CTS2 and CTR_2_: Ct value in the scion and rootstock of the graft-inoculated at DAT 180, respectively.

c SI: Scion infected percentage (%).

d LT: Las transmission (%).

To confirm the efficacy of antibiotics at different concentrations against Las bacterium, four antibiotics (PEN, ZS, VA and KSM) were evaluated at three concentrations (10 mg/L, 100 mg/L and 1000 mg/L). No significant concentration effects were found on antibacterial activity against Las bacterium (Table 5).

**Table 5:** Efficacy of antibiotics at different concentrations against Las bacteria.

Antibiotics	Concs (mg/L)	Scion survival (%)	Scion grown rate (%)	Scion infected (%)	Las transmission (%)	Ct value in scion	Ct value in rootstock
VA	10	90.9	26.8±18.6 a	60.4±8.3 a	45.0±11.2 a	32.6±1.6 a	33.1±1.9 a
(Effective)	100	95.0	36.3±12.9 a	68.3±2.4 a	40.9±9.3 a	32.4±1.2 a	33.4±1.8 a
	1000	100.0	42.5±30.7 a	74.4±10.4 a	46.4±5.1 a	32.7±1.9 a	33.7±0.4 a
ZS	10	75.0	14.2±0.5 a	55.0±5.4 a	35.7±10.3 a	32.0±2.6 a	32.2±2.7 a
(Effective)	100	75.0	18.7±8.8 a	50.0±1.0 a	31.2±8.8 a	33.6±0.9 a	34.5±0.3 a
	1000	71.4	21.8±4.4 a	70.8±5.9 a	30.5±11.4 a	32.5±0.2 a	34.4±0.9 a
KSM	10	95.8	89.5±14.7 a	79.0±0.2 a	66.6±13.6 a	28.6±0.2 a	28.5±2.5 a
(Noneffective)	100	91.7	82.5±17.7 a	80.0±0.2 a	68.3±11.8 a	30.0±0.5 a	27.8±1.9 a
	1000	90.8	86.4±18.3 a	75.2±0.3 a	65.4±15.8 a	29.8±0.4 a	28.5±0.8 a
PEN	10	75.0	68.0±14.1 a	0.0±0.0 a	0.0±0.0 a	38.3±0.4 a	38.7±0.8 a
(Highly	100	96.4	73.2±2.5 a	0.0±0.0 a	0.0±0.0 a	39.7±0.1 a	39.6±0.2 a
effective)	1000	84.6	71.4±10.3 a	0.0±0.0 a	0.0±0.0 a	39.2±1.2 a	40.0±0.8 a

Transmission efficiency of Las along with scion survival (%) and scion infection (%) and the Ct value in the scion and rootstock in the inoculated plants when grafted with Las-infected scions (Ct value = 23.7±1.3) and treated with antibiotics at concentrations of 10 mg/L, 100 mg/L and 1000 mg/L.

## Discussion

Nearly 40 antibiotics have been screened to control bacterial diseases of fruit trees and to limit the contamination of micropropagation and plant tissue cultures for>50 years. However, less than 10 antibiotics have been used commercially and, of those, only streptomycin and tetracycline have been knowingly applied to fruit trees (43). Although effective chemical treatments for commercial application have yet to be developed for the control of citrus HLB, we have demonstrated some chemical compounds that reduce Las titer in infected trees [9], [30], [31]. Earlier experiments, using the graft-based chemotherapy screening method, determined a mixture of penicillin and streptomycin could suppress or eliminate Las [9], [31]. Continuing to use this method, thirty-one antibiotics based on the suggestions from scientists around the world and the manufacturers' suggestion were evaluated, each at a single concentration, for their efficacy against Las and phytotoxicity to citrus. Principal component (PCA) and hierarchical cluster (HCA) analysis indicated that these antibiotics were clustered into 3 groups based on their antibacterial activities: *i*) highly effective (AMP, CAR, PEN, CEF, RIM and SDX), which produced a lower percentage of infected scions and rootstocks, and lower bacterial titers in the treated inoculated plants; *ii*) partly effective (CHL, VA, SMZ, CYS, KAN, SPT, ZS, HYG, RIX, CIP, STZ and RIF); and *iii*) non-effective (AMK, PMB, KSM, COL, STR, VAN, CIN, GAT, NEO, TOB and LIN) along with controls (water, DMSO and ethanol), which have the highest percentages of Las-infection and amounts of bacterial titers.

Within group I, three beta-lactam antibiotics, AMP, PEN and CAR, were shown to completely eliminate Las bacteria from the Las-infected scions. The graft-inoculated plants had no HLB-like symptoms and no Las bacteria were transmitted from the scions to the rootstocks (Table 2). Beta-lactam antibiotics inhibit the growth of sensitive bacteria by inactivating enzymes located in the bacterial cell membrane, known as penicillin binding proteins, which are involved in cell wall synthesis [32]. Cheema et al. (1986) also reported that penicillin combined with carbendazim eliminated Las [33]. We previously reported that penicillin G potassium [9], [30] or ampicillin [31] at 1,000 mg/L eliminated ‘*Ca.* L. asiaticus’ in infected periwinkle and citrus [29], [30]. In this experiment, PEN was reduced to 10 mg/L and still suppressed Las titer levels above the cutoff of Ct 36, thus eliminating Las infection in both the scion and the rootstock, and was relatively non-phytotoxic (∼70% of scions lived but their growth was reduced). Applications of water-soluble penicillin G salts have been patented for increasing plant size or vigor (U.S. Patent US2749230) and reducing the time required for sugarcane to reach normal maturity (U.S. Patent US3897239). Similar results in efficacy and phytotoxicity were present with CAR. CEF that belongs to the cephalosporins, a subgroup of β-lactams, also reduced Las titer in scions and the percentage of infected scions and rootstocks but was the least effective out of all β-lactams. Although β-lactam antibiotics has not been approved by the Environmental Protection Agency (EPA) for crop plants, the effectiveness of penicillin for control of Las and its ability to promote plant growth may merit further research and regulatory consideration for emergency and restricted usage.

Sulfonamide antibiotics (SDX, STZ and SMZ) are organic sulfur compounds containing the radical -SO_2_NH_2_ (the amides of sulfonic acids). Its molecular structure is similar to p-aminobenzoic acid (PABA), a substrate of the enzyme dihydropteroate synthetase required for the synthesis of tetrahydrofolic acid in bacteria [34], [35]. Sulfonamide, derived from sulfanilamide, inhibits bacterial growth by interfering with the metabolic processes that require PABA. Results presented here showed that SDX and STZ at the concentration of 100 mg/L effectively suppressed Las (27.8 and 47.9 scion infected % and 10 and 21.1 Las transmission %, respectively); whereas, SMZ was slightly less effective with higher percentages of scions infected (81.3%) and increased Las transmission (28.8%) (Table 2). Of the three sulfonamides, SDX was highly effective at reducing Las titer and thus considered a group I antibiotic (Table 3). All three compounds had moderate phytotoxicity with the scions producing fewer flushes. In addition to inhibiting bacterial growth, SDX and STZ have been reported to alter the root morphology and functionality in carrot, lettuce, alfalfa, and barley [36]. The use of sulfonamides may alter the productivity of the treated plant and thus further studies are necessary to understand the effect of sulfonamide antibiotics on citrus.

Rifamycins are a group of antibiotics, including RIM, RIF and RIX, that are synthesized either naturally by the bacterium, *Amycolatopsis mediterranei*, or produced artificially. Antibacterial activity of rifamycin results from the inhibition of bacterial DNA-dependent RNA synthesis [37]. Rifamycin is commonly used to prevent bacterial contamination when isolating or growing fungi [38], [39]. In addition, it kills intracellular bacteria, and thus is quite effective against mycobacterial infections [37]. Our results indicated that RIM, RIF and RIX at the concentration of 50 mg/L reduced the percentage of infected scions with 20.5%, 40.7% and 52.4% in average, respectively (Table 2). Las were averagely transmitted to 16.1%, 45.8% and 50.0% of the scion-treated grafted rootstocks, respectively. In addition, RIF had little to no phytotoxicity with 91.7% of scions surviving and 85.4% growing well. Out of these three antibiotics, RIM is the more promising candidate for future testing against Las. Typically, rifamycins have a bacteriostatic effect against Gram-negative bacteria [40]. Thus, additionally testing is needed to determine the duration of RIF efficacy and if reapplication of rifamycin over time is necessary to continue the suppression of Las.

Tetracycline is the only antibiotic approved for injection into the trunks of palm and elm trees to treat lethal yellow diseases caused by phytoplasmas [41]. During the 1970s, tetracycline was evaluated by direct injection into the trunks of HLB-affected citrus trees in South Africa, China and Indonesia [23]. Significant reduction of symptoms was observed in the treated trees; however, this treatment at that time was not commercially feasible because tetracycline was bacteriostatic, and required repeated treatments each year. OXY and CHL were selected for screening and showed varying degrees of effectiveness against Las. In this study, OXY at the concentration of 100 mg/L effectively reduced Las titer but was phytotoxic to citrus. Although OXY was removed from all statistical analyses due to lack of living scions, future experiments using reduced concentrations may be warranted. On the other hand, CHL at 30 mg/L was less effective than OXY but was not phytotoxic. These results indicated that OXY and CHL should be screened using a range of concentrations to determine if a tetracycline could be used to effectively manage Las infections while reducing the phytotoxicity on citrus. OXY has already been approved for the management of bacterial spot in stone fruits, apple and pear fire blight [42], [43].

Aminoglycosides inhibit bacterial protein synthesis through irreversible binding to the 30S bacterial ribosome [44]. More results presented here indicated that six out of nine aminoglycoside antibiotics (AMK, GAT, KSM, STR, NEO and TOB) were not effective in suppressing Las; KAN, SPT and HYG were partially effective. Antibacterial efficacy can be influenced by multiple factors such as bacterial responsiveness, physicochemical environment at the site of infection and interaction with the host [45], [46]. Per results of the initial screening, these six antibiotics are not ideal candidates for further testing against Las but it may be possible to increase their efficacy by using different concentrations and using multiple antibiotics at once. In this report, 80% of the scions soaked in STR at 100 mg/L were positive for Las and 65% of the grafted rootstocks became infected (Table 2). In our previous reports, the combination of beta-lactam antibiotics (PEN) and aminoglycoside antibiotic (STR) eliminated Las in HLB-affected citrus plants in the greenhouse and field [9]. PEN and STR are two separate classes of antibiotics and thus have different mechanisms of action. STR was only effective when combined with PEN indicating there is a synergistic effect against Las bacterium [47].

Two other antibiotics screened were zhongshengmycin (ZS) and validamycin A (VA). These are of particular interest as that they are broad-spectrum, mildly toxic agro-specific antibiotics. These compounds provide effective control against a variety of bacterial and fungal diseases [48], [49]. ZS, a N-glycoside antibiotic produced by strain of *Streptomyces*, has been used to control both bacterial and fungal diseases of melon, vegetables, and fruit trees including citrus in China [50]. VA is also produced by another strain of *Streptomyces* and has been used to control rice sheath blight [51]. Our results indicated that ZS and VA suppressed Las but they were not as effective as the beta-lactam antibiotics (Table 2). Although ZS and VA did not eliminate Las completely, they are strong candidates for future HLB control due to their classification as an agro-specific antibiotics, low phytotoxicity, and ability to reduce Las titer.

Of the remaining compounds screened, CYS and CIP were classified into group II, partly effective (Table 3). Although in this study these compounds did not eradicate Las, their effectiveness could be increased by using different concentrations.

To be a viable candidate for control of citrus HLB, the antibiotic should be characterized as: (i) active inside of the plant; (ii) tolerant of oxidation, UV irradiation, rainfall, and high temperatures; (iii) non-phytotoxic to citrus; and (iv) having a low or non-detectable rate of resistant pathogens. Las bacterium is an uncultured bacterium which resides in the citrus phloem [1], [4]; therefore, its minimum inhibitory concentration (MIC) of the antibiotics cannot be tested in pure culture. Although the citrus graft-based method requires a longer period of time for evaluation, it is a direct method for screening molecules for the control of HLB while assessing their phytotoxicity to citrus. Additional concentrations and application methods will be further tested including those compounds in the partly effective with less phytotoxicity and highly effective with greater phytotoxicity groups. One potential application method already in use at commercial orchards is to mix antibiotic powders in a large volume of water to a concentration of 50–200 parts per million (ppm) and apply to tree canopies [43].

Due to the dire nature of needing a control method, the effectiveness of compounds in Group I will be evaluated in a further field trial. Meanwhile, additional greenhouse testing can determine if the treatment results of Group II compounds can be increased while maintaining low levels of phytotoxicity. The screened effective antibiotics, especially agricultural antibiotics, have potential applications to rescue the invaluable citrus plants infected by HLB, to obtain pathogen-free citrus nurseries in the “Citrus Variety Improvement Program”, and to protect the home-garden citrus tree thereby, minimizing a potential pathogen reservoir. In addition to identifying the best HLB control compound with high antibacterial activity and low phytotoxicity, the effective method of application remains to be determined for citrus trees. Although trunk-injection of antibiotics is labor intensive, it decreases environmental exposure of antibiotics; and thereby, reduces the potential for unwanted impacts.

The public concern opposed to antibiotic use on plants is that spraying antibiotics in the open environment and over large expanses of land might increase the emergence of antibiotic-resistant bacteria. In our previous studies, three OTUs (Operational Taxonomic Units) were identified as oxytetracycline resistant genes but no penicillin resistant genes emerged after trunk-injected in the field for over one year [52]. Streptomycin and tetracycline resistance genes have been shown to be often carried on the same large plasmid in orchard bacteria but when the plasmid with these genes was transformed into *Escherichia coli*, the new host was only resistant to tetracycline and not to streptomycin or the other antibiotics [53].

This research may assist regulatory agencies in evaluating the potential for applying antibiotic treatments in the future to larger grove settings. It would be of great value to cure Las-infected citrus trees; however, development and/or refinement of application techniques will be necessary prior to commercial application. Careful comparison of costs and benefits of injection treatments versus other control measures will be needed to determine whether antibiotic treatments are economically feasible, and if the efficacy and phytotoxicity of the compound merits expense and deregulation for commercial use.
